# The U&I study: study protocol for a feasibility randomised controlled trial of a pre-cognitive behavioural therapy digital ‘informed choice’ intervention to improve attitudes towards uptake and implementation of CBT for psychosis

**DOI:** 10.1186/s13063-018-3023-7

**Published:** 2018-11-20

**Authors:** Kathryn Greenwood, Katie Alford, Ian O’Leary, Emmanuelle Peters, Amy Hardy, Kate Cavanagh, Andy P. Field, Richard de Visser, David Fowler, Matthew Davies, Alexandra Papamichail, Philippa Garety

**Affiliations:** 1 R&D Department, Sussex Partnership NHS Foundation Trust, Sussex Education Centre, Millview Hospital Site, Nevill Avenue, Hove, BN3 7HZ UK; 2 School of Psychology, University of Sussex, Pevensey Building, Falmer, Brighton, East Sussex BN1 9RP UK; 3 Brighton and Sussex Medical School, University of Sussex, Falmer, Brighton, BN1 9RP UK; 4 Department of Psychology, Institute of Psychiatry, Psychology and Neuroscience, Kings College London, De Crespigny Park, Denmark Hill, London, SE5 8AF UK; 5 PICuP Clinic, South London and Maudsley NHS Foundation Trust, Maudsley Hospital, Denmark Hill, London, SE5 8NZ UK; 6 South London and Maudsley NHS Foundation Trust, Bethlem Royal Hospital, Monks Orchard Road, Beckenham, Kent BR3 3BX UK

**Keywords:** Pre-CBT, Psychosis, Psychoeducation, Informed choice, Decision aid, Intervention, Feasibility, Pilot, Implementation

## Abstract

**Background:**

At least 40% of people with psychosis have persistent distressing symptoms despite optimal medication treatment. Cognitive behaviour therapy for psychosis (CBTp) is the only NICE-recommended individual therapy for psychosis, with effects on symptoms, distress and quality of life. Yet <10% of service-users receive it and 94% of trusts struggle to provide it. Of those offered it, 22–43% refuse or do not attend. We have developed a new pre-CBTp informed choice intervention to address knowledge and attitudes that influence uptake and implementation and now want to test it in a feasibility trial.

**Methods:**

The design is a two-arm, feasibility randomised controlled trial (RCT), with 1:1 randomisation, stratified by participant group and site. Participants are 40 psychosis patients and 40 clinicians, who are ambivalent towards uptake or implementation of CBTp. Sites are community and inpatient services in Sussex and London. The intervention is a pre-CBT digital psychoeducation intervention designed to address identified knowledge and attitudinal barriers to uptake and implementation of CBTp, incorporating behaviour change mechanisms, and supported by animated introductory, patient and clinician stories. The comparator is the NHS choices website for CBT. The primary aim is to assess clinical feasibility (recruitment, randomisation, acceptability, use, delivery, outcome measurement, retention). A secondary aim is a preliminary evaluation of efficacy. Outcomes will be assessed at baseline, post intervention, and one-month follow-up (blind to treatment arm). The primary efficacy outcome is likelihood of offering/taking up CBTp. Secondary outcomes include knowledge and attitudes towards CBTp, illness perceptions, empowerment, psychological wellbeing (patients only) and CBTp implementation (clinicians only). Use of the intervention and CBT behaviours during the follow-up period will be recorded and captured in a feedback questionnaire. Use, acceptability and experience of outcome assessment will be explored in qualitative interviews with participants (*n* = 6 per group). The efficacy evaluation will report descriptive data, key model parameters and 95% highest probability density intervals in a Bayesian growth model.

**Discussion:**

This is the first feasibility trial of a digital ‘informed choice’ decision aid for the implementation of CBTp. If the trial proves feasible and demonstrates preliminary evidence of efficacy, a large multi-site trial will be warranted.

**Trial registration:**

ISRCTN registry, ISRCTN53107879. Registered prospectively on 2 August 2017.

**Electronic supplementary material:**

The online version of this article (10.1186/s13063-018-3023-7) contains supplementary material, which is available to authorized users.

## Background

People who experience psychosis comprise approximately 1% of the UK population (approximately 600,000 people). At least 40% have persistent distressing symptoms, and poor recovery, despite optimal medication treatment. Apart from the significant distress to patients, they make use of considerable NHS inpatient and community resources. The cost has been estimated at £6.7 billion [1]. Cognitive behavioural therapy for psychosis (CBTp) is the only NICE-recommended individual therapy for psychosis that is cost-effective [2–4] with effects in reducing hospitalisation, symptoms and distress, improving quality of life and social functioning [5–9]. Yet < 10% of service-users receive it and 94% of trusts struggle to provide it [10, 11] with similar problems and levels of implementation occurring internationally [12]. Moreover, of those offered it, 22–43% refuse or do not attend [13]^.^ There is a dearth of research into barriers to implementation in CBTp [14] and research on uptake is also limited.

In terms of CBTp delivery, diffusion, dissemination and implementation are progressively more active steps in translating the evidence into practice [15]. Dissemination is the process of distributing information to stakeholders, for example via guidelines, manuals and training, but it is insufficient to produce sustainable changes in practitioner behaviour [16]. Implementation of evidence-based health care presents a serious challenge and training transfer into routine practice is notoriously problematic [17]. A number of strategies are indicated to enhance implementation but no simple solution has been identified [18, 19].

These implementation strategies incorporate six key processes of planning, educating, financing, restructuring, quality control and policy content [19]. In relation to CBTp, identified barriers to implementation are consistent with these processes and include issues with workforce planning, shortages of trained staff and supervisors, limits in funding and in organisational support. However, at an individual level, limited knowledge and perceived relevance of psychological therapies, such as CBTp, and pessimistic attitudes on the part of clinicians also play a part [20, 21]. Specifically, implementation requires some change in the ‘adopter’ needs, motivations, values, goals and skills towards CBTp, again emphasising clinician knowledge, beliefs and attitudes. These cognitive factors might include perceived relative advantage of CBTp compared with other approaches, compatibility with one’s own views, health and illness perceptions, complexity and ease of use of the intervention and observability, or seeing it as achievable, watching it in action and knowing that it works [18, 22, 23]. Indeed, the Theory of Planned Behaviour requires that implementation is desirable, associated with positive attitudes and perceived to be within behavioural control [24], while Normalisation Process Theory proposes that implementation requires coherence with clinical practice, cognitive participation and engagement with the processes of intervention delivery, collective action of individuals, teams and services to support delivery, and reflexive monitoring of the impact of intervention delivery processes [25]. The knowledge and attitudes of clinicians are thus argued to be key to implementation of psychological interventions in psychosis [26–28] with some arguing that these attitudinal barriers may be at least as important to address as therapeutic skill training [29].

Significant steps have been made in addressing barriers at the organisational, financial, training and competency level with the advent of Improving Access to Psychological Therapies in Severe Mental Illness (Psychosis) (IAPT-SMI) national pilot sites and agreed competencies for therapy delivery. Indeed, Jolley and colleagues reported a threefold increase in accepted referrals for NICE-recommended CBTp in a single year, when supported by strong organisational readiness, established referral pathways, referral monitoring and managerial support, ring-fenced funding for trained therapists, and embedding within specialist psychosis service where staff were knowledgeable about the difficulties faced in psychosis and about appropriate treatments and engagement approaches [30]. Newer brief, intensive interventions may also help to improve implementation, as they can be targeted and delivered in shorter time frames [31–33]. However, there is still much to be done to increase implementation. The London IAPT-SMI pilot delivered to only 300 patients, from an estimated pool of 3500 eligible participants, and brief intensive interventions are not yet embedded within routine practice.

Only two small-scale qualitative studies in the UK have investigated implementation at the individual clinician level in terms of knowledge and beliefs, finding that barriers exist in terms of beliefs about who is appropriate for CBTp, who will accept it, who will benefit and who should deliver it [20, 21]. This pattern is repeated internationally, with low priority given to CBTp by clinicians in a recent German study, influenced by lack of availability of general and specialist CBTp training and limited normalising illness beliefs [34]. Kimhy et al. also found considerable knowledge gaps concerning the evidence base for CBTp in the US, even in psychiatry and psychology training directors [35].

However, improved implementation is only half of the picture. Uptake of interventions in general [36–39], and of CBTp in particular, may be hampered by health, illness and treatment perceptions [40–42]. Illness perceptions in psychosis patients also impact on quality of life [36–38]. Specific interventions, based on changing illness perceptions, have been effective in promoting adherence to and outcomes from physical health interventions and have emphasised the need to tailor these interventions to health beliefs [43, 44]. Brief interventions that encourage more adaptive beliefs about illness and informed choice regarding psychological treatment in psychosis are clearly needed [13, 45, 46], both to encourage uptake and to improve outcomes.

Hence, problems exist both in implementation by clinicians and uptake by service-users, with significant potential impact on prolonging distress and disability. NICE have emphasised the need for research to consider knowledge and beliefs when developing and evaluating interventions aimed at behaviour change among individuals or populations [2]. Interventions drawing on implementation science and health perceptions may provide the mechanism, while information technologies may offer the means of delivery, being easy to implement in routine care. Interactive web-based interventions hold the potential to bridge the ‘quality chasm’ enabling the delivery of effective standardised interactive materials, in accessible, flexible, cost and time-efficient formats, blending a range of approaches, that can be flexible to the knowledge, beliefs and behaviours of individuals, incorporating motivational and behaviour change techniques, and being readily refined through use and feedback [47–53].

In summary, CBTp is an effective intervention for distressing symptoms of psychosis yet few service-users receive it. Despite the potential for increased capacity to deliver CBTp through training, this has not translated well into increased implementation or uptake. This is in part hampered by gaps in knowledge and pessimistic attitudes. There has been little research aimed at addressing these gaps.

### Research question

Is a randomised controlled trial (RCT) of a pre-CBTp digital ‘informed choice’ intervention to improve knowledge, beliefs and behaviours of clinicians and service-users feasible to be implemented?

### Aims and objectives

This is the first feasibility RCT to develop and test a pre-CBT digital ‘informed choice’ psychoeducation intervention, designed to address identified knowledge and attitudinal barriers to uptake by psychosis patients and offers by clinicians of CBTp, and ultimately to improve implementation. The primary research aim is to explore trial methods, clinician and patient acceptability of the interventions and outcome measures, and to provide data to estimate the parameters required to design a future RCT. The primary objectives are as follows:

1. To determine the number of participants who accept referral and randomisation (benchmark set at 80% of target for progression);
2. To evaluate the appropriateness of eligibility criteria (ambivalence towards CBTp) by determining feasible eligibility and recruitment rates at each site;
3. To assess retention through post-intervention and one-month follow-up rates (benchmark set at minimum 80% retention in line with previous studies);
4. To assess acceptability of the intervention, and factors influencing this, through usage data in the follow-up period, feedback questionnaire responses, and qualitative interviews;
5. To assess the acceptability and feasibility of the outcome measures as methods to measure efficacy of the intervention within a future trial.

Secondary objectives will be:

1. To measure and describe key outcomes post intervention and at one-month-follow up (e.g. completion rates, likelihood of offering/referring or taking CBT). Descriptive statistics will be used to quantify dropout and differences in mean responses on key outcome measures over time within each arm. Contingent upon the similarity of protocol for a future trial to this feasibility trial, Bayesian models may be fit to determine plausible estimates for the effect of the trial under both informative and uninformative priors.

We will not report significance tests as the feasibility RCT was not designed or powered to test hypotheses or to detect change. We have selected the NHS choices information about CBT as the comparison intervention as this is the next best alternative online resource about CBT that is routinely available for use by NHS clinicians and the public.

## Method

### Design

**Fig. 1:**
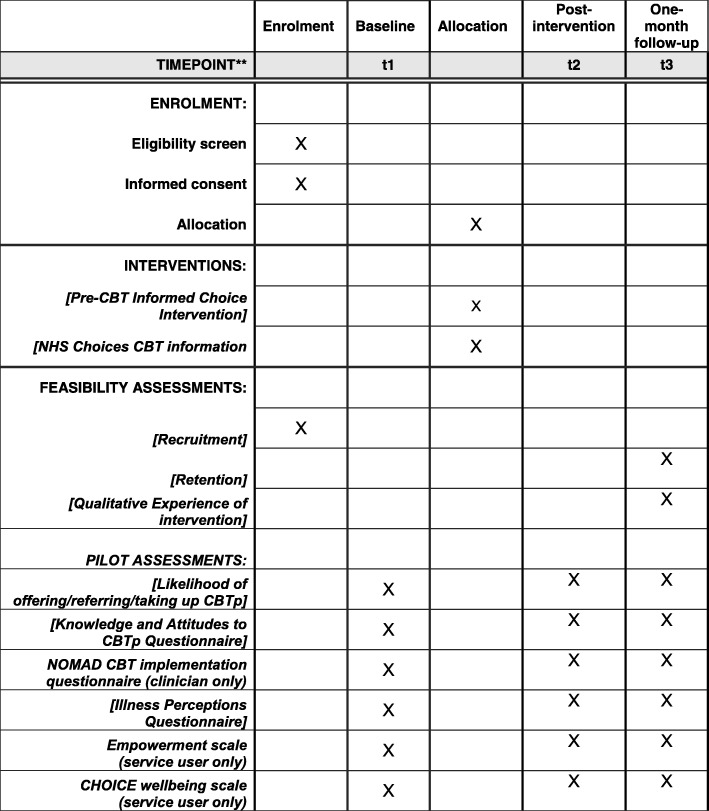
Figure for Standard Protocol Items (SPIRIT) for an interventional trial of a pre-CBT digital ‘informed choice’ psychoeducation intervention for clinicians who work with psychosis and their psychosis service users (the U&I study): schedule of enrolment, interventions, and assessments

The design is a two-arm, multicentre, longitudinal, feasibility RCT, with 1:1 block randomisation and varying block size, stratified by participant group (clinician or patient) and site (London or Sussex). Participants will be randomised by Kings College London Clinical Trials Unit, independently from the trial team, using an online randomisation system. Randomisation will be initiated by the research assistant immediately after the baseline assessment and before intervention delivery. Allocation will be confirmed by email. Baseline and one-month follow-up data will be collected blind to allocation to ensure unbiased data collection. Post-intervention data will not be collected blind to allocation as these data are collected in a single session, immediately after intervention delivery. Participants will not be blind and will be reminded of the blind procedure at the start of the one-month follow-up, in order to reduce the risk that they reveal their allocation, and blind breaks will be recorded. For reporting the feasibility of the RCT, the CONSORT (Consolidated Standards of Reporting Trials; http://www.consort-statement.org/) extension to randomised pilot and feasibility trials statement will be followed [54]. For the protocol, the SPIRIT (Standard Protocol Items: Recommendations for Interventional Trials) Figure and Checklist are provided in this paper see: Fig. 1 and Additional file 1.

### Setting

The setting will be secondary NHS community and inpatient services in two geographically distinct NHS Trusts: Sussex Partnership NHS Foundation Trust (rural and urban) and South London and Maudsley NHS Foundation Trust (urban inner city).

### Participants

Participants will be 40 current psychosis patients and 40 clinicians working with people with psychosis, aged 16–65 years, who have expressed ambivalence towards uptake or implementation of CBTp.

Inclusion criteria are:

- A current psychotic disorder diagnosis (F20-F29 ICD-10 diagnoses) as evidenced by clinical notes and/or discussion with lead mental health professional (patients only);
- Receiving treatment from secondary mental health services for psychosis or working as a clinician in a secondary mental health service that delivers treatments for psychosis;
- Age 16–65 years;
- Holding ambivalent views regarding CBTp, (defined as a score of ≤ 7 on a 10-point Likert scale for likelihood of taking up CBTp (patients) or offering/referring for CBTp (clinicians).

Exclusion criteria:

- Lack of capacity to give informed consent;
- Insufficient grasp of the English language to enable questionnaire completion;
- Cognitive impairment or learning disability which precludes engagement with the study materials.

### Trial flowchart

**Fig. 2:**
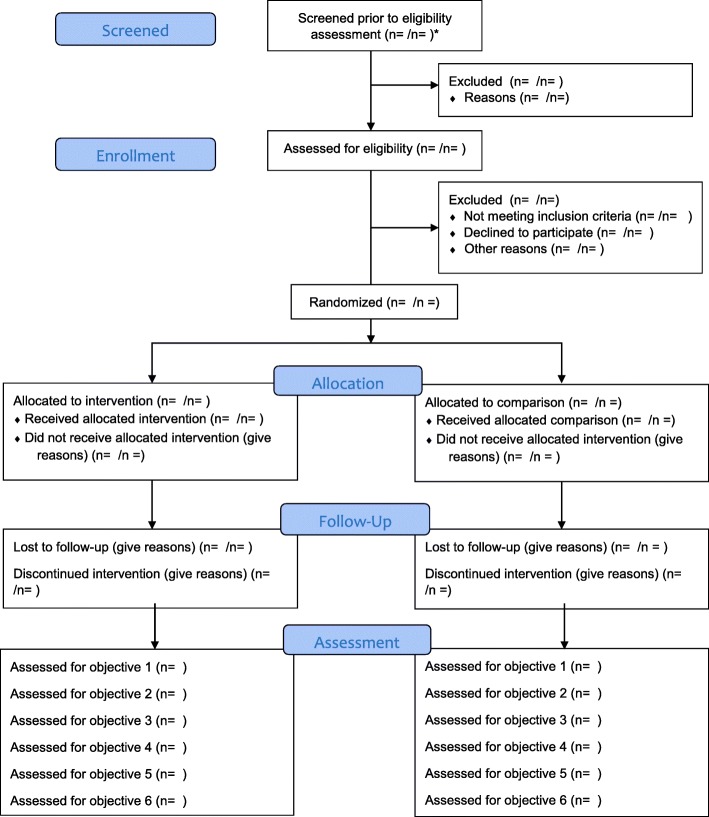
Trial flowchart for the U&I study feasibility RCT protocol

Figure 2 illustrates the trial flowchart.

### Intervention development

The intervention was developed over three phases of user-centred design and research process. The purpose of these phases was: (1) to identify a rich set of issues affecting uptake and implementation through a qualitative study; (2) to identify the largest and most significant of these issues in two large quantitative questionnaire studies; and (3) to develop a broad set of intervention components and to select the most important of these through workshops and a Delphi consultation.

First, qualitative interviews were conducted with 37 clinicians and 27 patients with psychosis in order to determine the nature of knowledge and attitudinal barriers to uptake and implementation of CBTp. The interviews were transcribed and analysed thematically; the content was used to develop the two knowledge and attitudes towards CBTp questionnaires (described below). In phase 2, these questionnaires were given to 206 clinicians and 206 patients; the data were used to identify the most significant factors influencing uptake and implementation. In phase 3, a user-centred design approach, and workshops with patients, clinicians and the research team, led by an industrial designer and Uscreates, a health design agency, developed and tested the design concepts, components and functions. The initial stories and content were informed by the rich qualitative data, questionnaire data and workshops. A Delphi consultation with clinicians, patients, CBT trainers and experts in digital therapy (*n* = 12) reached consensus on components of the final intervention.

### Intervention

The pre-CBT digital ‘informed-choice’ psychoeducation intervention is a website with information, animated stories and interactive elements, designed to address identified knowledge and attitudinal barriers to uptake and implementation of CBTp in clinicians and patients. It provides honest, balanced and hopeful information, incorporating behaviour change mechanisms derived from the theory of planned behaviour, and models of health perceptions, and is supported by animated introductory, patient and clinician stories. An interactive goals section aims to encourage motivation, while the stories, allow patients and clinicians to compare themselves to others with similar experiences, and to see CBTp in action, and hear how it works. The intervention includes sections on what CBTp is; how and whether it works; what it can help with and for whom; how it compares to alternative treatments; what CBTp might be like, and advice on preparing to start CBTp. It also contains handouts and sections for family and friends, and for the whole clinical team, which aim to promote a cohesive and supportive social and clinical network for the implementation of CBTp. The animated stories are an especially important component. They incorporate key messages derived from the earlier stages of intervention development. They can be watched either as complete stories or in sections that illustrate the three main topics: What is CBTp? What will it be like? And Is CBTp for me?

The order of accessing the materials is fixed and participants are encouraged to visit the different sections in order, following a journey represented by a sailing boat, through the intervention, with a route map and arrows that indicate the direction of travel. Although the participant can select their own ‘goals’ within the intervention and print these, no data are saved, thus avoiding patients’ concerns about personal data security. Although the intervention is designed for both patients and clinicians, patients felt strongly that they should have access to the same information as clinicians, while clinicians felt that the intervention should be geared towards the needs of patients; as such, only a single intervention platform was developed.

**Fig. 3:**
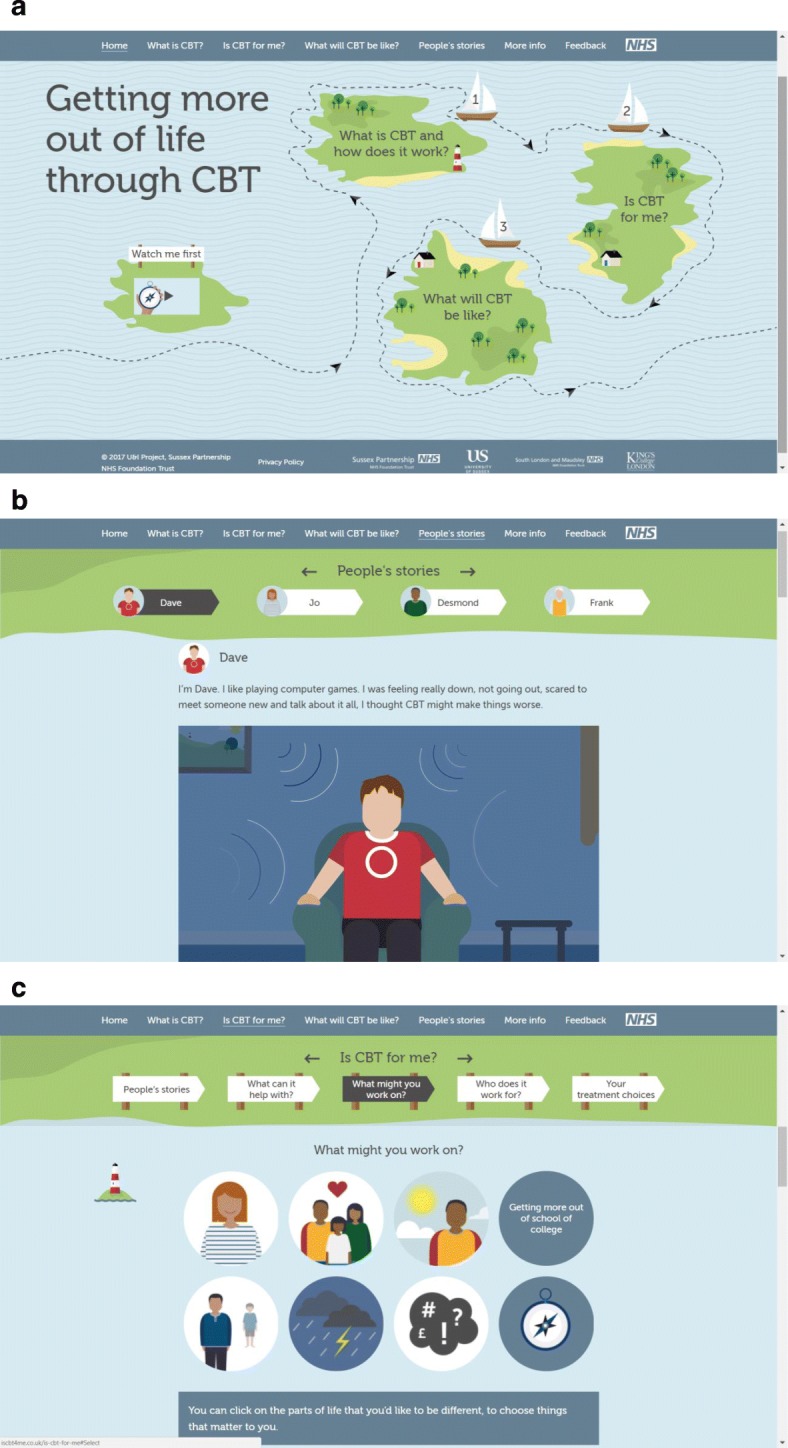
The intervention website. **a** The home page. **b** A patient’s story. **c** An interactive page

The intervention will be delivered during a manualised introductory session of up to 1-h duration, by a trained research assistant. Access to the intervention will be available for the subsequent month by computer or iPad, before follow-up. No specific instructions will be given for how or when participants should access the intervention, but overall usage data will be recorded. Images of the intervention are shown in Fig. 3.

The manual comprises a three-page document, which describes how to introduce the digital intervention to a clinician or a service user. The introduction explains: (1) how the intervention has been developed; (2) the prominent features of the intervention that are of relevance for each type of user (clinician or service user); and (3) the way in which each type of user might use the intervention in the subsequent month. Subsequent sections of the manual describe which sections of the intervention to visit, in which order, for how long, and which features and functions should be demonstrated and content emphasised in each section. Finally, the manual directs the research assistant to provide the user with an information sheet with the website address and space to record their experiences of use in the subsequent month.

Each research assistant who delivers the intervention will be provided with the manual and the intervention and will be required to practice delivery with and ask questions of a trained colleague to ensure confidence and competence before first use.

### Comparator

The active comparator intervention is the CBT section of the NHS choices website. It controls for attention and intention and represents best-case routine care in relation to CBT decision-making. It contains written text in five sections: How CBT works; Uses for CBT; What happens during a session; Pros and Cons of CBT; and Finding a therapist. It also includes a short, written user story. It will be presented in an equivalent session of up to 1 h, with a printed summary sheet provided as a handout, alongside the link to the website for use in the subsequent month.

All routine care and interventions will be permitted throughout the duration of the study.

### Assessments of outcome

Please refer to Fig. 1 (SPIRIT figure) for details of assessment at each visit. The sample sizes have been determined by pragmatic means to establish the feasibility of study and intervention procedures.

### Primary feasibility outcomes

The primary purpose is to assess the feasibility of the interventions in terms of acceptability, usability and accessibility, and to examine the feasibility of methods of a future trial including measures, recruitment procedures and trial processes. This will be done through monitoring using the study CONSORT diagram, through system analytics on use of the digital intervention during the one-month period, through the user feedback questionnaire collected at the end of one month, and through a qualitative interview with a sub-sample of those receiving the intervention (*n* = 6 per group) at the end of one month. The primary feasibility outcomes are:

1. Feasibility of eligibility criteria, recruitment and retention within the NHS (numbers referred, recruited, consented, randomised and retained in the trial) during the trial recruitment period of August 2017 to May 2018;
2. Feasibility of use, accessibility and acceptability of the intervention and the outcome measures in the NHS, measured using feedback questionnaire responses regarding use of the intervention; activity data on use of the intervention during the follow-up period (average number of pages visited per participant), qualitative interviews with six patients and six clinicians (three each in Sussex and London) regarding their experience of using the intervention, the selection of outcome measures and participation in the trial; September 2017 to May 2018.

### Efficacy evaluation outcomes

Participants will complete the following measures at (1) baseline, (2) immediately after introduction to the intervention, and (3) at one-month follow-up.

1. Likelihood of offering/referring or taking up CBT will be measured using a single Likert scale (0–10) for how likely the participant is, in general, to offer/refer psychosis patients for CBT, if this were available, or how likely the participant is to take up CBTp if it were offered today, each rated from not at all (0) to extremely likely (10);
2. Knowledge and attitudes towards CBTp will be measured using mean factor score for two new questionnaires developed during earlier phases of the current study. The clinician questionnaire comprises 27 items, which form three factors (pro CBT content, supportive CBT culture, confidence in the value of CBT to patients), all with high internal consistency (α = 0.87, 0.82 and 0.71, respectively) and predictive validity for likelihood of referring/offering CBTp (robust estimate of correlation = 0.45, 0.53 and 0.32, respectively). The patient questionnaire comprises 35 items, which form four factors (positive attitudes towards CBTp, personal barriers, doubt regarding efficacy and fear related to CBTp), each with good internal consistency (α = 0.94, 0.83, 0.76 and 0.82, respectively) and predictive validity (robust estimate of correlation = 0.50, 0.46 and 0.54, respectively), except fear which was not a good predictor of uptake (robust estimate of correlation = 0.26). In each questionnaire, items are scored on a 7-point Likert scale from disagree strongly to agree strongly;
3. Illness perceptions will be measured using the mean factor scores for the modified illness perceptions questionnaire [55] which has good internal consistency (α = 0.70) and forms three factors (cure/control, timeline, causes). The cure/control and timeline factors have good internal reliability and test–retest reliability (cure/control α = 0.09/0.09 and timeline α = 0.7/0.06). Individual items are scored on a 5-point Likert scale from strongly agree to strongly disagree;
4. Attitudes and behaviours towards CBTp implementation will be measured using mean scores for five constructs of the NOMAD tool [56] adapted for CBTp implementation (clinicians only). The tool includes three items that assess normalisation of CBTp on a 0–10 Likert scale from ‘still feels very new’ to ‘feels completely familiar’, and 20 items that assess the four core constructs of coherence of CBTp with standard care, cognitive participation, collective action and reflexive monitoring, each rated on a 5-point Likert scale from strongly agree to strongly disagree;
5. Psychological wellbeing will be measured using the CHOICE short-form measure of psychological wellbeing (patients only). This scale has been produced by our research group and has good psychometric properties including internal consistency (α = 0.83), test–retest reliability (ICC = 0.73), convergent validity (*r* = 0.52 with quality of life; − 0.58 with mood) and sensitivity to change (*t* = − 0.41) [57]. The short form used in the current study has been widely used including in the national IAPT-SMI pilot sites in the UK [30], and includes 11 items, and one personal goal item, each rated on a 0–10 Likert scale from ‘worst’ to ‘best’. The total mean score will be used;
6. Empowerment will be measured using the mean factor scores for the Rogers’ Empowerment scale (patients only) [58] which has good internal consistency (α = 0.82) and factor structure with five factors: self-esteem (α = 0.82); perceived power (α = 0.59); optimism and control over the future (α = 0.45); community activism/autonomy (α = 0.59); and righteous anger (α = 0.64), with good convergent (hope/recovery *r* = 0.67, empowerment *r* = 0.45) and predictive validity (psychosis symptoms *r* = − 0.39). The scale includes 28 items measured on a 4-point Likert scale from strongly agree to strongly disagree;
7. CBT-related activities in the preceding month will be based on interview and case notes screen (at one-month follow-up).

The interview regarding CBT-related activities is part of a larger interview to explore attitudes to and use of the intervention during the follow-up period. The whole interview takes approximately 10 min.

## Procedure

Clinician and patient participants will be identified through research staff presentations at team meetings, clinics, training events and following email and telephone contact with staff.

Clinician participants will be approached by a research assistant and patient participants by their lead clinician. All interested potential participants will be provided with the study information sheet and will have the opportunity to discuss the study in a meeting or telephone conversation with a research assistant who is trained in taking informed consent and in Good Clinical Practice. Following informed consent, participants will complete the basic demographic information and baseline questionnaire measures. They will then be randomised to either the intervention or comparison condition. The intervention or comparison condition will be provided according to a manualised protocol as part of a single session lasting up to 1 h, after which participants will also complete the same questionnaires in the post-intervention assessment in the same session.

Participants in the intervention condition will be provided with access to the online intervention for the subsequent month. Where a patient participant does not have access to a computer, they will be provided with an iPad, locked to the intervention site, to enable access in the subsequent month. Usage data will be tracked through Google Analytics and will be stored safely and confidentially; data willonly be accessible to the researchers via a password. Usage data will be stored in an encoded format that prevents tampering. Comparison participants will be provided with the NHS choices website link and handout. This website is publicly available. After one month (− 2/+ 8 weeks), each participant will complete the same questionnaires in a follow-up assessment, which will be conducted by a research assistant who is blind to randomisation arm, in person or by telephone. All participants in the intervention arm will also complete a brief feedback questionnaire on their use of the intervention.

A small subsample of six patients and six clinicians who receive the informed choice intervention materials will also undertake a brief semi-structured interview with a research assistant to explore their experience of using the intervention materials and to provide feedback to further refine the intervention.

### Analysis plan

Descriptive data will be used to report referrals, recruitment, retention, use of the intervention and CBTp-related behaviour. Audio-recorded qualitative interviews will be transcribed and themes will be extracted using Interpretative Thematic Analysis. Themes will be translated into recommendations for refining the intervention, trial procedures, outcome measures and protocol in preparation for a future RCT, if feasibility and preliminary efficacy data are promising. There will be a preliminary examination of the data after 10 participants per group have been recruited. If, at that stage, the intervention and protocol are regarded as sound and needing no significant refinement, the study will proceed to recruit the full 40 participants per group. If there is need for further refinement, the study will allow for successive refinement of the protocol with the next 10 participants per group until the trial team are satisfied that the final protocol is optimal. If the protocol changes during data collection descriptive statistics will be reported (where feasible) by timepoints at which protocol changes were made. These will not be used to inform a subsequent trial, but only to describe what happened in the current trial. If the protocol does not change during data collection and, therefore, a subsequent trial would adhere to the current protocol, descriptive statistics will be reported; a Bayesian growth model will also be fit to the data, blind to study arm, treating change over time as a random effect within participants and randomisation arm as a time-invariant fixed effect. 95% highest probability density (HPD) intervals will be used to estimate plausible values of the key model parameters (notably the parameter quantifying the difference between the arms in the rate of change of the likelihood of taking up/referring to CBT). Bayesian growth model parameters are estimated using an iterative process (Markov chain Monte Carlo [MCMC]) and missing values are estimated as unknown parameters [59] in a process akin to multiple imputation [60]. To check the influence of priors of the model estimates, model fit with both informative and uninformative priors will be compared.

### Data management and security

Data storage and confidentiality will be ensured, in line with the Data Protection Act 1998 and the NHS Code of Practice for Confidentiality. All personally identifiable data will be kept separately from anonymised research data and all data will be stored in secure password-protected files, available only to the study team, on Sussex Partnership NHS Foundation Trust protected access shared drives and in locked filing cabinets. Study data will be identified using a participant identification number (ID). This ID will be linked to the participant’s name in a password protected link file. Audio-recording equipment will be used to record qualitative interviews regarding the experiences of taking part in the intervention and the trial. These audio files, named with the unique participant identifier, will be stored as computer files on secure NHS servers in an anonymised and encrypted form.

The digital intervention will record how patients and clinicians use the intervention over the month that they have access, including which pages are accessed and how frequently. Only data about use of the package will be stored. No personal data will be stored. Usage data will be tracked through Google Analytics and stored safely and confidentially in an encoded and password-protected format that prevents tampering and that is only accessible to the researchers.

### Data quality

Data quality will be ensured by close monitoring and routine auditing for accuracy. The main feasibility and pilot outcome data will be checked for every participant by comparing the paper record with that on the database, once all possible assessments for each time point have been completed. An error rate of no more than 5% is acceptable. If an error rate > 5% is found, advice will be sought from the trial statistician regarding further data checking.

## Study governance

Sussex Partnership NHS Foundation Trust is the sponsor for this study. The trial has received a favourable ethical opinion from London-Dulwich research ethics committee (reference 15/LO/0041; IRAS number 176709). The trial will be conducted in compliance with the principles of the Declaration of Helsinki [61], the Medical Research Council Guidelines for Good Clinical Practice [62] and in accordance with all applicable regulatory requirements including the Research Governance Framework and the Mental Capacity Act 2005 [63]. The chief investigator will have overall responsibility for the trial dataset, supported by the trial oversight group. The sponsor and REC will be provided with direct access to source data and other documents if required for REC review and trial monitoring. The trial management group will meet approximately fortnightly to review trial conduct and a trial oversight group and patient and public involvement group will meet approximately every 3–6 months to oversee study conduct, analysis and outcomes, including recruitment and data completion. The trial oversight group will comprise members of the study team who are not involved with the day to day running of the study. They will approve the protocol and amendments and in this feasibility study will undertake DMEC duties. The trial is registered at ISRCTN (reference 14678860), a primary clinical trial registry recognised by WHO and ICMJE. Individual participants will have the right to withdraw from the trial at any time. Any serious adverse events will be reported to the trial oversight group. The trial may be prematurely discontinued by the sponsor, chief investigator or on the basis of new safety information or for other reasons by the oversight group, regulatory authority or Ethics Committee. The trial may also be prematurely discontinued due to lack of recruitment. If the study is prematurely discontinued, active participants will be informed and no further participant data will be collected.

### Public involvement

Patient and public involvement (PPI) has been central throughout the entire project. A patient co-applicant with lived experience of psychosis co-ordinated the PPI and with PPI members with psychosis, co-led on developing the research question, and collaborated and provided rich perspectives on the design, ethics, recruitment, delivery and dissemination strategy, part-funded by a Research Design Service PPI grant. Key impacts included the focus on patient uptake alongside implementation and the incorporation of feedback from those who have used the intervention to inform future users. They emphasised the potential of the intervention to raise awareness and understanding of CBTp and thus improve uptake, provide goals, offer hope for recovery and impact on important wellbeing outcomes, which were incorporated into the outcome measures. The PPI lead and a designated service-user researcher contributed to earlier phase data collection, qualitative analysis and questionnaire development. A study-specific advisory group co-facilitated with the PPI lead have advised on the intervention materials and interpretation of earlier phase outcomes. They will continue to meet approximately every three months to provide input to the study and steering group regarding recruitment, evaluation and dissemination of results.

### Dissemination

Results will be presented from different perspectives (patient, clinician, trainer) and to a range of audiences, through patient and public, and academic peer-reviewed journals. All study co-applicants and research assistants will contribute to all study publications; all study publications will be submitted for review to the National Institute for Health Research before publication.

## Discussion

This trial represents the initial feasibility RCT of the first pre-CBT informed choice intervention. The intervention has been designed following extensive qualitative and quantitative analysis and user-centred design research. It focusses on the needs, knowledge and attitudes of psychosis patients and clinicians, and is a psycho-educational and decision-aid tool to facilitate high quality, informed and collaborative discussions and choices regarding the implementation and uptake of CBT for psychosis. The study will explore feasibility, accessibility, acceptability and utility of the intervention in routine clinical practice in the NHS to inform the design for a future RCT. The careful assessment of feasibility will help to ensure effective future implementation within the NHS of the digital intervention [64].

We initially planned to develop two interventions: one for patients and one for clinicians, each addressing their unique needs, but during intervention development it became apparent that clinicians felt that the intervention should be focused on the needs of patients and patients felt equally that they should have access to the same information as clinicians. For these reasons, we developed a single intervention, but it will be important to understand the extent to which this truly addresses the needs and barriers for clinicians offering CBTp alongside those of patients.

The qualitative and quantitative feasibility feedback will enable us to determine how and when in a patient’s journey, or a clinician’s interaction with their patients, the intervention would be most appropriate and most valuable. It will also enable us to understand where and in what format the intervention might be most acceptable and accessible, bearing in mind both the strengths and limitations of an online intervention.

The inclusion of two sites each with a different ethos and organisational structure relating to CBTp will enable us to more readily generalise our learning to a range of NHS trusts and services.

Understanding and evaluating the ambivalence of clinicians and patients towards a NICE-recommended intervention requires sensitivity. An important outcome of the feasibility and pilot work will be to determine the best way to recruit to the study, capture ambivalence and evaluate outcomes in a study of this type. It may also provide some preliminary indication of the likely size of changes in knowledge, attitudes, intentions and behaviours towards CBTp.

The inclusion of measures of health perceptions and attitudes towards implementation will allow us to understand the factors that influence attitudes at baseline and will provide indicators of potential change mechanisms. Further indications of change mechanisms will be obtained from the qualitative feedback regarding which psychoeducation and behavioural change components were most used and most valued.

The final outcome from this study will be a robust protocol for a future RCT to test the effectiveness of the first digital ‘informed choice’ intervention to promote uptake and implementation of CBT for psychosis, as well as to explore purported mechanisms of action. The intervention has the potential to provide a valuable benchmark to enable high quality, collaborative discussions and offers of CBTp by clinicians, as well as empowering patients to make informed choices about uptake. If effective, this intervention or ‘therapeutic decision aid’ may pave the way for the development of additional decision aids to support informed choices about other psychological and non-pharmacological approaches, thus enabling these interventions to be better matched and targeted to patient needs and preferences.

### Trial status

Recruitment of participants commenced in August 2017 and will be open until May 2018. The date of first enrolment is August 2017.

## Additional file

Additional file 1: SPIRIT Checklist for the U&I Project. (DOC 121 kb)

## Abbreviations

CBTp: CBT for psychosis
CHOICE: CHoice of Outcome In Cbt for psychoses
CONSORT: Consolidated Standards of Reporting Trials
DMC: Data Monitoring Committee
GP: General practitioner
IAPT-SMI: Improving Access to Psychological Therapies in Severe Mental Illness
ICD-10: International Statistical Classification of Diseases and Related Health Problems 10th Revision
ICMJE: International Committee of Medical Journal Editors
IRAS: Integrated Research Application System
ISRCTN: International Standard Registered Clinical/soCial sTudy Number
NHS: National Health Service
NICE: National Institute for Health and Care Excellence
PPI: Patient and Public Involvement
RCT: Randomised controlled trial
REC: Research Ethics Committee
SPIRIT: Standard Protocol Items: Recommendations for Interventional Trials
WHO: World Health Organization
