# Correction to: The U&I study: study protocol for a feasibility randomised controlled trial of a pre-cognitive behavioural therapy digital ‘informed choice’ intervention to improve attitudes towards uptake and implementation of CBT for psychosis

**DOI:** 10.1186/s13063-018-3085-6

**Published:** 2018-12-12

**Authors:** Kathryn Greenwood, Katie Alford, Iain O’Leary, Emmanuelle Peters, Amy Hardy, Kate Cavanagh, Andy P. Field, Richard de Visser, David Fowler, Matthew Davies, Alexandra Papamichail, Philippa Garety

**Affiliations:** 1 R&D Department, Sussex Partnership NHS Foundation Trust, Sussex Education Centre, Millview Hospital Site, Nevill Avenue, Hove, BN3 7HZ UK; 2 School of Psychology, University of Sussex, Pevensey Building, Falmer, Brighton, East Sussex, BN1 9RP UK; 3 Brighton and Sussex Medical School, University of Sussex, Falmer, Brighton, BN1 9RP UK; 4 Department of Psychology, Institute of Psychiatry, Psychology and Neuroscience, Kings College London, De Crespigny Park, Denmark Hill, London, SE5 8AF UK; 5 PICuP Clinic, South London and Maudsley NHS Foundation Trust, Maudsley Hospital, Denmark Hill, London, SE5 8NZ UK; 6 South London and Maudsley NHS Foundation Trust, Bethlem Royal Hospital, Monks Orchard Road, Beckenham, Kent, BR3 3BX UK

**Correction to: Trials**

**https://doi.org/10.1186/s13063-018-3023-7**

Following publication of the original article [1], the authors reported a typing mistake in the spelling of author Iain O’Leary. The original article has been corrected.

In this Correction the incorrect and correct spellings are shown.

Incorrect spelling:

- Ian O’Leary

The correct spelling is:

- Iain O’Leary
